# Usefulness of case reports to improve medical knowledge regarding trigemino-cardiac reflex in skull base surgery

**DOI:** 10.1186/1752-1947-5-149

**Published:** 2011-04-15

**Authors:** Nora Sandu, Pooan Sadr-Eshkevari, Bernhard J Schaller

**Affiliations:** 1 Department of Neurosurgery, University of Paris, Paris, France; 2 Farzan Clinical Research Institute, Tehran, Iran; 3 Associate Editor, Journal of Medical Case Reports

## Abstract

We describe the discovery of the trigemino-cardiac reflex by Schaller in 1999 and the continued improvement of the knowledge about the trigemino-cardiac reflex involved in neurosurgery, especially in skull base surgery, during the past several years. The achieved medical progress could be gained only by the practical experience described by different case reports and later case series that have been published in several principal scientific journals. Additionally, we explain the scientific as well as clinical importance of the communication of the case reports on TCR. Special reference has been given to the validity of the case reports for new phenomena in clinical medicine.

## Editorial

Clinical case reports, which are often overlooked by mainstream scientific journals, represent an important, maybe even an exclusive, way of communicating new and unusual clinical findings in medicine to the scientific community. In addition, they are often the main source of new knowledge about rare clinical features in medicine, especially in surgery.

One such example is the discovery of the trigemino-cardiac reflex (TCR), a well-recognized brainstem reflex that consists of bradycardia, arterial hypotension, apnea and gastric hypermotility [1]. The current knowledge of this reflex is mainly based on a cornerstone article that introduced the topic into neurosurgery and skull base surgery in 1999 [1]. Clear proof of the reflex's existence was provided on a causal relationship basis, and the TCR arc was described to be constituted of trigeminal and cardioinhibitory vagus nerves as the afferent and efferent pathways. After this initial case series that suggested for the first time a definition of the TCR based on clinical and theoretical consideration, the subsequently published case reports could therefore focus on the differentiation between the peripheral and central stimulation [2]. These additional clinical data provided strong evidence of our hypothesis that the TCR triggered by peripheral stimulation (via the spinal nucleus of the trigeminal nerve to the Kölliger-Fuse nucleus) is different from the TCR triggered by central stimulation (via the nucleus of the solitary tract to the lateral parabrachial nucleus) [3].

It always represents a substantial problem to generalize from a few case reports to larger case series. Therefore, a critical reflexivity is absolutely needed, which we resolved by performing a causal relationship test in the initial TCR publication [1]. Thus, the TCR can be prevented by avoiding stimulation of the afferent pathway or by blocking the nerves that conduct the afferent impulses. Such a cause-and-effect relationship is evidence of a TCR, and the stimulation of the trigeminal root represents the afferent pathway. The few single-case reports that followed the initial report were therefore also important to rigorously test our first hypothesis of the existence of this newly described reflex. If just one such observational case does not fit completely with the proposition, the hypothesis should be considered generally not valid and therefore should be revised or even rejected. This was not the case with our hypothesis.

Case reports are especially suited for such further scientific investigations and theory building in clinical medicine because of their in-depth approach. For this reason, we additionally tested whether skull base operations other than for vestibular schwannoma also demonstrated an intraoperative occurrence of the TCR, and we finally found that the phenomenon can also be seen during transsphenoidal surgery [4,5] and during Janetta operations [6]. Because the issue of generalization has appeared in the (scientific) medical literature with regularity, we have conducted further research for TCR in different skull base approaches to facilitate this generalization. Such further case reports [7] and case series [8] also point out the functional importance of the TCR on the surgical outcome after skull base surgery. Focusing on hearing function in patients with vestibular schwannoma has demonstrated the hypotension owing to the TCR to be a negative prognostic factor for hearing preservation and postoperative tinnitus [3]. This fact points out that extreme or atypical cases often reveal more information because in them more basic mechanisms of the disease are activated. In addition, from both an understanding-oriented and an action-oriented perspective, it is often more important to clarify the deeper cause behind a given clinical picture and its consequences that describe only signs and symptoms of a disease and how frequently it occurs.

It is a frequent criticism of case-report-research that the results are not widely applicable in "real life". In particular, that criticism is underlined by presenting a well-constructed explanation of the difference between analytic generalization and statistical generalization: "In analytic generalization, previously developed theory is used as a template against which to compare the empirical results of the case study" [9]. This represents a well-established manner of work in clinical research. The appropriate manner of generalization does always assume that some *sample *of cases has been usually drawn from a larger "universe" of cases, but only one is presented. Thus, the incorrect terminology, such as "small sample size", arises as though a single-case study is considered unfairly only as a single respondent. For the TCR, however, we therefore published a review paper early in the research of the TCR [10] in which we analyzed roughly all cases published previously as a large collective and gave a detailed overview about the theory behind it and could also shed light on new investigations. This led researchers all over the world to become interested in this phenomenon and to popularize the topic of TCR.

Further case reports on the TCR inform about some risk factors for the intraoperative occurrence of the TCR [11-13]. Several factors have been postulated as predisposing patients to the TCR on the basis of a few clinical case reports. These factors include hypercapnia, hypoxemia, light anesthesia, high resting vagal tone in children, narcotics such a sufentanil and alfentanil, preoperative β-blockers and calcium channel blockers [3]. Cases such as the above-cited critical "follow-up cases" can have more importance in relation to the general clinical feature of the initial hypothesis than to merely represent a small sample of patients. If the initial hypothesis also applies in these "follow-up cases," it could also be expected to be applicable to a large range of patients. In this manner, by strategically selecting cases, one can arrive at the point at which cases allow generalization as a particular research methodology. Also, with more uniform considerations in reporting the TCR cases, case series and clinical trials, such statistical analyses of the literature would become easier and faster.

The TCR is now accepted all over the world on the basis of our initial definition and is considered one of the great problems in modern skull base surgery (see, for example, [14]). This example points out the outstanding importance of case reports to improve medical knowledge not only in past centuries but also nowadays. We hope that this story of success may inspire others to publish and especially to deeply analyze their special cases that deal with not yet published clinical features; this is one of the most important ways that medicine can advance. In addition, our experience and reports underline the importance of special journals such as the *Journal of Medical Case Reports *that deal only with case reports and of making great efforts to achieve a high quality of publication also in case reports and to stimulate authors to analyze their special cases.

## Competing interests

The authors declare that they have no competing interests.

## Authors' contributions

NS, PS and BS contributed equally to the writing of the manuscript. All authors have read and approved the final manuscript.
